# Is it worth turning a trigger into a joke? Humor as an emotion regulation strategy in remitted depression

**DOI:** 10.1002/brb3.1213

**Published:** 2019-01-21

**Authors:** Anna Braniecka, Małgorzata Hanć, Iwona Wołkowicz, Agnieszka Chrzczonowicz‐Stępień, Agnieszka Mikołajonek, Monika Lipiec

**Affiliations:** ^1^ Department of Psychology SWPS University of Social Sciences and Humanities Warsaw Poland; ^2^ Third Department of Psychiatry Institute of Psychiatry and Neurology Warsaw Poland

**Keywords:** depression, emotion regulation, humor, reappraisal, remission

## Abstract

**Aim:**

Humor has long been considered as an effective emotion regulation strategy for people vulnerable to depression, but empirical evidence in this area is scarce. To address this issue, we investigated the emotional consequences of humor in remitted depressed patients and compared them with the effects of positive reappraisal and spontaneous emotion regulation.

**Methods:**

Fifty‐five patients with remitted major depression took part in a laboratory computer experiment in which they were shown negative pictures twice. First, the patients simply viewed the pictures and rated their reactions. Second, they viewed each of the pictures according to instructions, which are to (a) use humor, (b) use positive reappraisal, or (c) simply view the pictures, and then, they again rated their reactions.

**Results:**

Humor was found to decrease negative emotions, increase positive emotions, and enhance the distance from adversity; it was more effective than spontaneous emotion regulation and similarly as effective as positive reappraisal. Humor was the most effortful form of emotion regulation. Patients were able to successfully produce humorous comments, and their failure to do so did not lead to worse emotional outcomes than regulating emotions spontaneously. The analyses also indicated that distancing mediates between using humor and the intensity of positive and negative emotions.

**Conclusions:**

Our findings provide preliminary empirical support for the idea that for individuals vulnerable to depression, humor can be an adaptive tool in dealing with negative responses to aversive events, and, thus, it may impair their potential of these events to trigger depressive episodes. Further studies in this area are warranted to determine the most adaptive forms of humor and analyze their effects in various depressogenic contexts.

Life is too important to be taken seriously. Oscar Wilde (1893)

## 1 INTRODUCTION

Depression is a highly recurrent mental disorder, with about 75% of recovered people having more additional episodes in their lifetime (Richards, 2011). According to the diathesis–stress approach (see Colodro‐Conde et al., 2018), depressive episodes develop as a result of an interaction between negative life events and individuals’ vulnerability to depression. When the vulnerability is high, which especially pertains to recurrent depressive disorder, the negative events required to trigger depression are typically less serious. Therefore, the experience of moderate stress is usually sufficient to trigger the first depressive episode, and from that point, an individual becomes increasingly susceptible to depression activated by relatively mild forms of adversity (Kendler, Thornton, & Gardner, 2001). Growing evidence shows that vulnerability to depression is closely related to using maladaptive emotion regulation strategies, which increases the likelihood of negative emotional experience escalating and leading to relapse or recurrence (see Joormann & Stanton, 2016). By contrast, adaptive emotion regulation strategies are indicated to improve emotional responses to adversity and, thus, reduce the risk of depression onset (Compare, Zarbo, Shonin, Van Gordon, & Marconi, 2014). The existing literature demonstrates that such strategies as acceptance, reappraisal, self‐compassion, and distraction are particularly effective in both current and remitted depression (Joormann & Stanton, 2016; Visted, Vøllestad, Nielsen, & Schanche, 2018). The objective of the present study is to investigate one promising but thus far unexamined emotion regulation strategy in recurrent depression—humor.

Individuals at risk for depression have long been encouraged to deal with adversity by using humor, and this can be observed in psychological training, self‐help books, and psychotherapy (see Martin, 2007). It should be stressed, however, that a large discrepancy exists between the application of humor in depressed people and scientific research. Nevertheless, what we know about humor's basic mechanisms seems encouraging from the perspective of its potentially preventive impact. Indeed, it appears that the mechanisms of humor may counteract main vulnerability factors to depression, such as impaired experiencing of positive emotions (Fava, 1999), increased ruminating and deficient cognitive inhibition of negative information (Joormann & D'Avanzato, 2010; Watkins & Nolen‐Hoeksema, 2014), and reduced distancing from adverse experiences (Teasdale et al., 2002). Humor seems to have the potential to undermine these inclinations by respectively inducing intense positive emotions, distracting from dwelling on negative thoughts, and providing a less serious interpretation of negative situations, leading to a more distant perspective on adversity (see Samson & Gross, 2014).

More specifically, humor serves as a powerful source of intense positive emotions, such as amusement, exhilaration, or mirth and therefore has the potential to undo lingering negative affect with its physiological consequences (Fredrickson, Mancuso, Branigan, & Tugade, 2000). It was shown that the frequent experience of positive emotions (including amusement) in the aftermath of crises broadens individuals’ attention and thinking, which, in turn, improves coping and buffers against depressive symptoms (Fredrickson, 2003). Indeed, enhancing the experienced positivity in formerly depressed individuals is regarded as a crucial component of well‐being therapy (Fava, 1999), which has been shown to substantially improve residual symptoms, especially anhedonia, and reduce the risk of relapse (e.g., Fava, 2016; Seligman, Rashid, & Parks, 2006). In line with this approach, it is increasingly emphasized that enhancing positive emotions, although left unaddressed in conventional treatment, is an essential component of effective preventive interventions in recurrent depression (see Santos et al., 2013). Interestingly, some evidence shows that positive, humor‐based experiences can provide emotional benefits that are comparable or even superior to those of aerobic exercise (Szabo, 2003). In an experiment conducted on a student population in this study, the participants were asked to either view humorous videos, run at a self‐selected pace, or watch neutral videos; each of the activities was scheduled for 20 min over three sessions. Both humor and physical exercise were found to be more effective than watching neutral materials in reducing anxiety and improving emotional well‐being, psychological distress, and fatigue. The anxiety‐lowering effects of humor were also found to be greater than those of physical exercise.

Another relevant mechanism of humor is distracting from the negative meaning of distressing situations; this is closely related to the cognitive demands of humorous materials and has been proven to be an effective tool for relieving depressed individuals’ low mood, which is mostly caused by decreasing rumination (Nolen‐Hoeksema, Wisco, & Lyubomirsky, 2008). Distraction is considered particularly adaptive for people at risk for depression because in the face of a potentially triggering negative event, it uses the limited processing capacity in the working memory and thus enables disengagement from mood‐congruent materials (Van Dillen & Koole, 2007). In consequence, negative emotional responses can be prevented from escalating into a full syndrome of depressive episode. In accordance with this view, experimental research on humor shows that directing attention from negative stimuli into mood‐incongruent, humorous materials attenuates the negative mood to a larger extent than do equally positive nonhumorous materials, and this is due to the greater cognitive demands and distraction involved in humor processing (Strick, Holland, van Baaren, & van Knippenberg, 2009).

The mechanism of humor that is considered particularly relevant from the perspective of emotion regulation is distancing form adversity (see Samson & Gross, 2014). In accordance with the perspective‐taking approach, humor dramatically changes the meaning of a negative situation into a less serious and less threatening one (Lefcourt et al., 1995). This is believed to involve radical cognitive changes and corresponding modifications in emotional responding, typically described as a cognitive–affective shift (Lefcourt, 2001). In consequence, an individual can successfully distance himself/herself from a negative situation and appraise its meaning form a less distressing point of view. The adaptive effect of humor‐based distance was evidenced, among others, in an experimental research on the self‐threat paradigm, in which it was demonstrated that humor led to the appraisal of adversity in a less disturbing manner—in both a harmful and acceptable way. Furthermore, clinical studies on metacognitive awareness in recurrent depression evidenced that remitted patients tend to respond to negative experiences with a substantially reduced mental distance, which was shown to be an important vulnerability factor for relapse (Watkins, Teasdale, & Williams, 2000). Moreover, it was found that enhancing patients’ capacity to distance from adverse stimuli and to experience them within a broad perspective increases their resilience (Teasdale et al., 2002).

Although clinical studies on humor and recurrent major depression are limited, existing reports on these are promising. The research of Falkenberg, Jarmuzek, Bartels, and Wild (2011a) on psychiatric inpatients with depression demonstrated that despite mild cognitive deficits in processing humorous materials (Uekermann et al., 2008), depressed individuals are generally susceptible to humor and do not differ from healthy people in rating humorous stimuli as being funny. The observed impairment in patients’ use of coping humor and their reduced inclinations toward humor behavior have been suggested not to limit their potential to develop humor skills but rather indicate the need to broaden this population's repertoire of coping tools with humor‐based strategies (Falkenberg, Jarmuzek et al., 2011a).

In accordance with this proposition, some preliminary evidence shows that training depressed inpatients in general humor skills may provide important benefits. For instance, Falkenberg, Buchkremer, Bartels, and Wild (2011b) implemented a manual‐based humor training, developed by McGhee (2010), in a group of patients hospitalized with major depression. During eight consecutive meetings that consisted of both theoretical information and practical application, the participants attempted to develop and enhance key humor skills, such as adopting a playful attitude, finding humor in everyday life, or laughing at oneself. Following the training, the patients displayed short‐term emotional improvement, as well as a subjective increase in their motivation and capacity to use humor in distressing situations (Falkenberg, Buchkremer et al., 2011b). Similar results were reported by Konradt, Hirsch, Jonitz, and Junglas (2013), who demonstrated that the application of McGhee's humor training in older inpatients with major depression may provide important benefits. More precisely, although both experimental (with humor training) and control groups (with no humor training) were found to show improvements in their depressive symptoms, suicidal thoughts, and states of cheerfulness and bad mood, only the participants of the humor group displayed beneficial changes in life satisfaction and state seriousness (Konradt et al., 2013).

Taken together, the existing empirical evidence on emotion in recurrent major depression seems to indicate that humor can be considered a valuable emotion regulation strategy, especially in remitted depressed patients. However, it is important to note that the use of humor in adverse situations is commonly recognized as challenging and effortful. Indeed, several lines of experimental research show that the humorous regulation of negative emotions requires substantial cognitive resources (Strick et al., 2009) and is reported to be difficult (Samson & Gross, 2012). For example, the humorous reappraisal of negative pictures was reported by student participants as significantly more difficult than serious reappraisal, and they were less successful in its use (Samson, Glassco, Lee, & Gross, 2014). This result seems particularly relevant in view of the increasing evidence that recurrent depressive disorder involves difficulties in stable emotion regulation (see Visted et al., 2018) and deficient cognitive processing of humorous materials (Uekermann et al., 2008). If so, given the great effort required from depressed individuals to apply humor and the high risk of failure involved, an important question arises on whether the benefits of turning a trigger into a joke can be significant enough to justify its costs.

The objective of this study is to contribute to filling this gap by investigating how humor applied in the face of adverse stimuli by remitted depressed patients affects their negative emotions, positive emotions, and distance from adversity. According to the perspective‐taking view (Lefcourt et al., 1995), humor is particularly effective in regulating negative emotions when its content is related to an emotion‐inducing stimulus, providing a way for individuals to reappraise the negative situation from a new and less threatening point of view. Therefore, in the present study, we focused on the form of humor that addresses negative situations and changes their meaning, which is described as humorous reappraisal.

We also aimed to compare humor with other generally adaptive emotion regulation strategies, such as positive reappraisal, as well as with patients’ spontaneous emotion regulation. We presumed that both positive and humorous reappraisal would be more beneficial than regulating negative emotions spontaneously. This expectation is based on substantial empirical evidence that remitted individuals with major depression show stable negative biases and deficits in cognitive control, leading to the habitual use of maladaptive emotion regulation strategies (see Joormann & Stanton, 2016).

Additionally, we hypothesized that although humorous reappraisal would require more effort, it would be more beneficial than positive reappraisal. This expectation is consistent with the result of previous experiments conducted on nonclinical populations, which indicate that although both humor and cognitive reappraisal lead to emotional improvements, these changes are greater in humor (Kugler & Kuhbandner, 2015; Samson et al., 2014). Despite the fact that both these strategies are based on reframing the negative meaning of a situation and increasing the experienced positivity, humor appears to be more powerful because it induces a much stronger positive affect in the form of exhilaration, amusement, or mirth, and it entails a cognitive–affective shift related to providing a greater change of perspective. Positive reappraisal seems to change negative meaning in a simpler way and results in much milder, if any, positive emotions.

Moreover, the goal of this research was to test the hypothesis that the key mechanism through which humor positively affects negative emotions in remitted depression is distancing from adversity. As such, we aimed to analyze its mediating role between humorous reappraisal and patients’ emotional outcomes. As already mentioned, humor has long been considered an important way of changing perspective from seriousness to play, which enables disengagement from disruptive adversity‐related thoughts, feelings or images (see Martin, 2007). In remitted depression, mental distancing has been shown to lead to particularly adaptive and buffering consequences. Specifically, clinical research on major depression demonstrated that patients who approach adversity from a distanced perspective exhibit lower levels of depressive thought accessibility and negative emotions than individuals who were more immersed in their negative experiences (Kross, Gard, Deldin, Clifton, & Ayduk, 2012). Moreover, increased distance from negative experiences was found to predict a reduced risk of depression onset during remission (Teasdale et al., 2002).

Although humor is often regarded as a generally adaptive way of dealing with adversity, it has been emphasized that many types or forms of humor exist, and each has its own mental health consequences (see Martin, 2007). Some of these forms, especially those related to a cheerful outlook in life or to laughing at adversity in a constructive way, are regarded as powerful strategies for dealing with negative experiences, whereas other forms of humor, such as sarcasm, mockery, or ridicule, are traditionally interpreted as dysfunctional (Vaillant, 2000). There have been considerable studies on individual differences in the use of humor, demonstrating that there are four distinct humor styles: two adaptive and two maladaptive ones (see Martin, Puhlik‐Doris, Larsen, Gray, & Weir, 2003). There is numerous, mostly correlational, evidence that adaptive styles of humor (affiliative and self‐enhancing), as opposed to maladaptive ones (aggressive and self‐defeating), are positively related to various mental health components, such as self‐esteem, optimism/pessimism, depressive symptoms, or anxiety (e.g., Martin, 2007). For instance, Rnic, Dozois, and Martin (2016) evidenced that decreased adaptive and increased maladaptive humor styles are associated with greater depressogenic cognitive distortions. The analyses also demonstrated that the less frequent use of self‐enhancing humor mediates the relationship between intense cognitive distortions and enhanced depressive symptoms, whereas the habitual use of self‐defeating humor, although primarily aimed at coping with distorted thinking in distressing social situations, was found to backfire and result in enhanced dysphoria (Rnic et al., 2016).

Studies investigating humor from an emotion regulation perspective refer to its heterogeneity according to a traditional approach, already suggested by Freud (1928), and distinguish between two forms of humor: positive (benevolent, good natured, nonhostile) and negative (aggressive, mean spirited, disparaging) (see Samson & Gross, 2014). Evidence supporting this perspective comes mostly from experimental research performed on nonclinical populations. The results indicate that positive humor is a more effective emotion regulation strategy than negative humor in terms of decreasing negative and increasing positive emotions (Samson & Gross, 2012), and that positive humor, compared with cognitive reappraisal, results in a greater reduction of negative affect and improves later memory for emotion‐eliciting information (Kugler & Kuhnbandner, 2015).

Altogether, we predicted (a) the positive consequences of humor reappraisal as an emotion regulation strategy in remitted major depression, (b) the greater effort required when using humor and the larger number of failures involved than when positive reappraisal is used, (c) the greater benefits from humor than from positive reappraisal and spontaneous emotion regulation, and (d) the mediating effect of distancing from adversity in the relation between the use of humor and emotional outcomes. We also aimed to examine the potentially adverse effect of failing to produce humor, which, in view of patients’ susceptibility toward defeat stress (Metalsky & Joiner, 1993), might have worse consequences than their spontaneous responses. In addition, according to the view that different forms of humor can be both adaptive and maladaptive (see Samson & Gross, 2014), we analyzed the content of humorous comments and determined the applied form of humor.

## 2 METHOD

### 2.1 Participants

#### 2.1.1 Power analysis

Calculation of the required sample size was performed in G*Power software, version 3.1.9.2 (RRID:SCR_013726). Data were to be analyzed with the use of repeated measures ANOVA and test main effects and within–between interaction. The analysis was used to detect the medium effect size even if correlations between repeated measures were weak. In accordance with Cohen's *f* effect size measure, a medium effect size takes the value of 0.25. It has been assumed that because of the random order of conditions for each participant, weak correlations between measures in terms of Pearson correlation coefficient *r *= 0.10 shall be established. Between‐subject factor divided the sample into two groups; there were three repeated measurements (because of three different conditions). To achieve statistical power of 0.80 with 0.05 level of significance, collecting data from at least 48 participants was needed. In case there were any errors in computer procedure, 55 patients were examined. The results for one participant were excluded because the results were below 3 *SD* from the sample's mean for two dependent variables.

#### 2.1.2 The sample

The final sample consisted of 54 outpatients ranging from 19 to 60 years of age. Participants were recruited from outpatient psychiatric clinics, where they regularly attended a mental health service. The basic inclusion criterion was a diagnosis of remission, after a depressive episode, made by a psychiatrist and confirmed with a structured clinical interview for DSM IV (SCID I; First, Spitzer, Gibbon, & Williams, 2002) administered by a trained interviewer who was blind to the results of the psychiatric interview. An additional inclusion criterion was a BDI‐II (Beck, Steer, & Brown, 1996) score of 16 or below, which indicates no more than mild severity of depressive symptoms (Smarr & Keefer, 2011). Exclusion criteria were (a) history of mania or psychosis and (b) current psychoactive substance use, eating disorders, anxiety disorders, intellectual disability, nervous system damage, pregnancy, and suicidal ideations. The sample characteristics are presented in Table 1. The majority of participants (79.63%) were medicated (Table 2).

**Table 1:** Summary of sample characteristics

	Frequency (%)	Mean (*SD*)	Statistics
		(*n* = 54)	
Demographic Information			
Age, years		40.85 (11.54)	
Gender			
Male	18 (33.33)		$\chi_{\left(1\right)}^{2}$ = 6.00 *p* < 0.05
Female	36 (66.67)		
Employment			
Employed/in education	24 (44.44)		$\chi_{\left(1\right)}^{2}$ = 0.67 *p* = ni.
Not employed	30 (55.56)		
Time in education, years		14.92 (2.94)	
Clinical Information			
BDI‐II		11.20 (4.89)	
Main diagnosis			
First depressive episode	16 (29.63)		$\chi_{\left(1\right)}^{2}$ = 8.96 *p* < 0.01
Recurrent depressive disorder	38 (70.37)		
Remission			
Full remission	22 (40.74)		$\chi_{\left(1\right)}^{2}$ = 1.85 *p* = ni.
Partial remission	32 (59.26)		
Length of remission			
About 1 month	27 (50.00)		$\chi_{\left(2\right)}^{2}$ = 8.11 *p* < 0.05
2–11 months	17 (31.48)		
1–2 years	10 (18.52)		
Lifetime number of depressive episodes		4.24 (4.24)	
Age of first onset, years		29.94 (13.49)	
Number of admissions		1.59 (2.80)	
Comorbidities			
No	37 (68.52)		$\chi_{\left(1\right)}^{2}$ = 7.41 *p* < 0.01
Yes	17 (31.48)		
Substance use disorders (full remission)	7 (12.96)		
Anxiety disorders	9 (16.67)		
Personality disorders	4 (7.41)		

Note

BDI‐II, Beck Depression Inventory, 2nd edition.

**Table 2:** Summary of sample medication

	Frequency (%)	
Unmedicated	11 (20.37)	$\chi_{\left(1\right)}^{2}$ = 21.41 *p* < 0.001
Medicated	43 (79.63)	
Antidepressants		
Amitriptyline	2 (3.70)	
Bupropion	1 (1.85)	
Citalopram	1 (1.85)	
Duloxetine	1 (1.85)	
Escitalopram	5 (9.26)	
Fluoxetine	8 (14.81)	
Mianserin	3 (5.55)	
Mirtazapine	6 (11.11)	
Paroxetine	3 (5.55)	
Reboxetine	1 (1.85)	
Sertraline	9 (16.67)	
Trazodone	5 (9.26)	
Venlafaxine	8 (14.81)	
Vortioxetine	1 (1.85)	
Antipsychotics		
Chlorprothixene	4 (7.41)	
Olanzapine	4 (7.41)	
Perazine	1 (1.85)	
Quetiapine	7 (12.96)	
Mood‐stabilizers		
Carbamazepine	2 (3.70)	
Lamotrigine	6 (11.11)	
Lithium	2 (3.70)	
Valproic acid	2 (3.70)	
Anxiolytics		
Buspirone	1 (1.85)	
Hydroxyzine	4 (7.41)	
Pregabalin	2 (3.70)	

#### 2.1.3 Ethics statement

This study was carried out in accordance with the principles laid down in the Helsinki Declaration of 1975, as revised in 2008, and was approved by the local ethics committee.

### 2.2 Procedure

Following psychiatric evaluation, each patient had an appointment with a clinician in order to complete the recruitment procedure (SCID I and BDI‐II fulfillment). The recruited patients were scheduled for the experimental part of the study, which took place within 1–3 days.

The experiment was based on a computer procedure developed by Samson et al. (2014) and commonly used in experimental studies on humor and emotion regulation. The procedure consisted of Phase 1 and Phase 2, during which participants observed a series of the same 28 negative pictures selected from the International Affective Picture System (IAPS; Lang, Bradley, & Cuthbert, 1997) which covered a variety of aversive stimuli (e.g., accidents, war scenes, sick people and animals). IAPS (RRID:SCR_016869) provides standardized sets of photographs accompanied by average ratings of valence, ranging from pleasant (9) to unpleasant (1), and arousal, ranging from calm (1) to excited (9). The pictures were selected to be negatively valenced (*M* = 2.8, *SD* = 0.76) and arousing (*M* = 5.2, *SD* = 0.9). This method for inducing negative emotions was aimed to reflect the typical triggers for depression which, in people who have a history depressive episodes, involves various relatively nonsevere stressful events (e.g., Kendler et al., 2001; Monroe et al., 2006).

In Phase 1, participants simply viewed the 28 pictures and after each one rated their responses (positive emotions, negative emotions, and distance from adversity). Each response was assessed on a single visual analogue scale (“How strong are your negative emotions at the moment?”; “How strong are your positive emotions at the moment?”; “How much distance to the scene, observed in the picture, do you keep?”) ranging from 0 (not at all) to 100 (as strong as possible/as much as possible). In Phase 2, participants viewed each picture a second time, under instructions either to (a) use humor, (b) use positive reappraisal, or (c) simply view. In the humor condition, participants were instructed to comment on the situation in a humorous way, to reconstruct its meaning as subjectively (not necessarily objectively) amusing. In the positive reappraisal condition, they were asked to generate positive comments that would reconstruct the meaning of the situation as benign, beneficial, or meaningful. In the spontaneous regulation condition, patients responded naturally; they simply viewed the picture for 5 s and then typed out what they saw on the screen (Supporting information Data S1).

In Phase 2, pictures were randomly assigned to each of the 3 conditions with 8 pictures attributed to each instruction (three 8‐trial blocks randomly presented for each participant). The structure of each trial was as follows. After an instructional slide asking to comment on the situation in a certain way, each picture was presented on the screen. After each picture, participants were asked whether they were able to produce the target comment (yes/sort of/no). Then, they typed out their humorous/positive comment or explained why they did not generate it. Next, participants rated their responses again and additionally reported the effort related to generating a comment, also on the VAS scale (“How much effort did you put into generating a comment?”) ranging from 0 (not at all) to 100 (as much as possible).

To facilitate the task, each block (except for the spontaneous regulation condition) was preceded by two pictures from Phase 1, with written examples for humor or for positive reappraisal, taken from the original procedure of Samson and Gross (2012) and developed after the pilot study. The entire procedure lasted 50–60 min.

All the humorous comments were analyzed in terms of the form of humor included in their content, according to the differentiation of Samson and Gross (2012) between positive and negative humor. Five trained coders, who were PhD psychology students, independently rated all the humorous comments (Kendall's *W* = 0.47). In accordance with Samson and Gross (2012), these coders rated a comment as representing positive humor when it was based on reappraising a picture in a benevolent way by expressing a sympathetic and tolerant amusement, focusing on imperfections of life and human nature or on absurdities of situations without depreciation or hostility. Negative humor was identified when a comment relied on malevolent reappraising by laughing at people or situations in a hostile, aggressive, or superior manner, expressing disdain and mocking others.

#### 2.2.1 Debriefing

After completing the study, each patient participated in a short debriefing session conducted by an experienced clinician who took reasonable steps to identify and minimize any harm to participants, as well as to provide them with detailed information about the meaning of the findings obtained. Overall, none of the participants reported being emotionally harmed in any way. Additionally, at the end of the experiment, participants were shown a series of positive pictures selected from the IAPS database.

### 2.3 Statistical analyses

Analyses were performed using SPSS version 23.0. (RRID:SCR_002865). Dependent variables were computed as difference scores: Phase 2 − Phase 1 (T2 − T1) in each condition (humorous reappraisal, positive reappraisal, spontaneous regulation). Trials in which participants did not generate a required comment (“no” response to the question “Have you produced a humorous/positive comment?”) were excluded from the main analyses. On average, patients complied with the instructions well; 79.07% of them succeeded in producing a required comment.

The tendency to produce a positive form of humor was computed for each participant as the total number of comments with positive humor, whereas the tendency to produce a negative form of humor was computed as the total number of comments with negative humor (produced in eight trials that were assigned to the humor condition). The number of comments based on positive humor was significantly higher (*M* = 4.87, *SD *= 2.00) than that based on negative humor (*M = *1.07, *SD *= 1.08), *Z *= −6.22, *p* < 0.001. As such, the asymmetry in the frequency of use of positive and negative humor would make further analyses, conducted separately for both forms of humor, invalid.

The form of humor and the use of medication were verified as possible covariates. However, no significant differences between patients with and without medication were found in terms of intensity of positive emotions: in humor, *t*(52) = −1.34, *p *> 0.05; positive reappraisal, *t*(52) = 0.98, *p *> 0.05; and spontaneous regulation, *t*(52) = 1.45, *p* > 0.05; in terms of negative emotions: in humor, *t*(52) = 0.07, *p* > 0.05; positive reappraisal, *t*(52) = −0.38, *p* > 0.05; and spontaneous regulation, *t*(52) = −0.74, *p* > 0.05; in terms of distance in humor, *t*(52) = 0.80, *p* > 0.05; positive reappraisal, *t*(52) = 0.14, *p* > 0.05; and spontaneous regulation, *t*(52) = −0.25, *p* > 0.05; nor in terms of effort in humor, *t*(52) = 1.29; positive reappraisal, *t*(52) = 1.16, *p* > 0.05; and spontaneous regulation, *t* (52) = 0.91, *p* > 0.05.

The tendency to generate a positive form of humor also did not correlate with the intensity of positive emotions: in humor, *r*(52) = 0.21, *p* > 0.05; positive reappraisal, *r*(52) = 0.15, *p *> 0.05; and spontaneous regulation, *r*(52) = 0.21, *p *> 0.05; with negative emotions in humor, *r*(52) = −0.07, *p *> 0.05; positive reappraisal, *r*(52) = −0.03, *p* > 0.05; and spontaneous regulation, *r*(52) = −0.14, *p* > 0.05; with distance in humor, *r*(52) = 0.13, *p* > 0.05; positive reappraisal, *r*(52) = −0.05, *p *> 0.05; and spontaneous regulation, *r*(52) = 0.17, *p* > 0.05; nor with effort in humor, *r*(52) = 0.19, *p* > 0.05; positive reappraisal, *r*(52) = 0.18, *p* > 0.05; and spontaneous regulation, *r*(52) = −0.05, *p *> 0.05. As neither the use of medication nor the type of produced humor was significantly related to all analyzed variables, both of these potential covariates have not been included in further statistical analyses.

Verification of the main hypotheses was performed using repeated measures ANOVA, with three conditions as within‐subjects variables, was computed with subsequent post hoc tests. The data that support the findings of this study are openly available in “Mendeley Data” at http://dx.doi.org/10.17632/cdjd7chk6g.1, v1 (RRID:SCR_015671).

## 3 RESULTS

**Figure 1:**
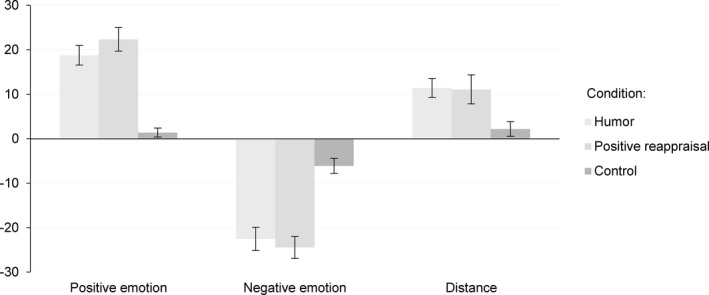
Average difference scores (T2 minus T1) with standard errors for positive emotions, negative emotions, and distance in three conditions: humor, positive reappraisal, and control

There were significant main effects of conditions for positive emotions, *F*(2, 106)* *= 34.32, *p* < 0.001, *η* ^2^ *^^*= 0.39, negative emotions, *F*(2,106) = 26.59, *p *< 0.001,* η* ^2^ = 0.33, and distance, *F*(1.79, 94.90) = 5.80, *p *< 0.001. Follow‐up *t* tests revealed that both humor (*M* = 18.8, *SD* = 16.7) and positive reappraisal (*M = *22.3, *SD* = 19.4) led to a higher level of positive emotions than spontaneous regulation condition (*M* = 1.4, *SD *= 7.4*)*, both *ps *< 0.001. Additionally, both humor (*M* = −22.5, *SD *= 19.2) and positive reappraisal (*M* = −24.4, *SD* = 18.1) led to a greater decrease in negative emotions than spontaneous regulation condition (*M* = −6.1*, SD *= 12.3), both *ps *< 0.001. Distance was also lower in spontaneous regulation (*M* = 2.2, *SD *= 12.1) than in the humor (*M* = 11.4, *SD *= 15.6), *p *< 0.01, and in the positive reappraisal condition (*M* = 11.1, *SD *= 24.0), *p < *0.05 (see Figure 1).

Generating humor was rated as more effortful (*M = *40.9, *SD* = 18.7) than positive reappraisal (*M* = 33.2, *SD* = 18.1), *F*(1,53)* *= 15.00, *p *< 0.001, *η* ^2 ^= 0.22. Consistently, comments were less completely successfully created (“yes” response) in the humor (*M* = 13.1%, *SD* = 11.3) than in positive reappraisal condition (*M* = 16.9%, *SD *= 11.6), *F*(1.53)* *= 11.41, *p *< 0.01, *η* ^2 ^= 0.18. Participants were more frequently partially successful (“sort of” response) in generating humor (*M* = 11.8%, *SD *= 10.54) than positive reappraisal (*M* = 9.0%, *SD* = 9.5), *F*(1.53)* *= 8.98, *p *< 0.01, *η* ^2 ^= 0.14, and they were more often unsuccessful (“no” response) in producing humor (*M* = 8.41%, *SD *= 8.3) than positive reappraisal (*M* = 5.17%, *SD *= 7.28), *F*(1,53)* *= 12.08, *p *< 0.01, *η* ^2 ^= 0.19. Overall, producing humor was less often successful (91.59% of all trials) than producing positive reappraisal (94.83% of all trials).

**Figure 2:**
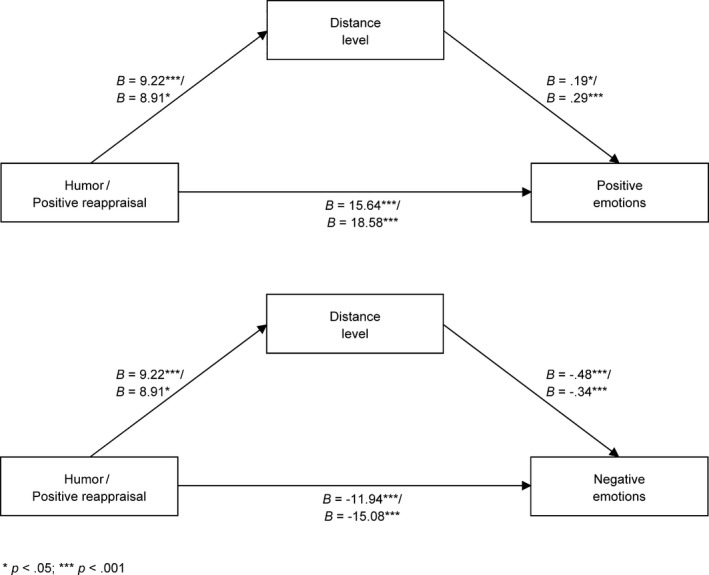
Distance and effort levels as mediators between the use of humor or positive reappraisal and intensity of positive and negative emotions

Distance and effort were analyzed as parallel mediators between the use of humor or positive reappraisal and positive/negative emotions. Four different models were analyzed: (a) humor—distance, effort—positive emotion; (b) humor—distance, effort—negative emotion; (c) positive reappraisal—distance, effort—positive emotion; and (d) positive reappraisal—distance, effort—negative emotion. The number of bootstrap samples for bias‐corrected bootstrap confidence intervals was 5,000. In all, four models’ explanatory variables were coded as binary variables, with a value of 0 for the control condition and of 1 for humor or the positive reappraisal condition. There were four significant mediation effects: a distance‐mediated relationship between humor and intensity of positive emotions, *B = *1.74, *SE *= 0.92 (0.36 ÷ 4.03), humor and intensity of negative emotions, *B *= −4.46, *SE *= 1.63 (−8.38 ÷ −1.85), positive reappraisal and intensity of positive emotions, *B* = 2.60, *SE *= 1.40 (0.49 ÷ 5.96), and positive reappraisal and intensity of negative emotions, *B *= −2.99, *SE *= 1.70 (−7.12 ÷ −0.48). The association between effort and positive emotions was nonsignificant. The association between effort and negative emotions was significant in the model with respect to humor, but the indirect effect was not significant, *B *= −0.01, *SE* = 0.68 (−1.43 ÷ 1.46). An illustration of mediation effects is presented in Figure 2.

We performed additional analyses verifying whether failing to use humorous reappraisal has worse consequences than spontaneous emotion regulation. The analysis was based only on trials in which participants did not create appropriate comments. There were no significant main effects of conditions for positive emotions, *F*(1.33, 29.27) = 3.34, *p *> 0.05, negative emotions, *F*(2,44) = 0.12, *p *> 0.05, or for distance, *F*(1.51, 33.17) = 1.32, *p *> 0.05, which indicates that failing to use humor (and positive reappraisal) did not lead to worse effects for emotions and distance than spontaneous emotion regulation.

We also verified whether depressive symptoms moderate the relation between the use of strategy and positive emotions, negative emotions, and distance. The intensity of depressive symptoms was included as a component of within–between interaction after the median‐split. The results of interaction tests for the level of depressive symptoms as moderator of the use of strategy and all explained variables were nonsignificant.

## 4 DISCUSSION

Is the use of humor as an emotion regulation tool for dealing with negative, potentially depressogenic events worth encouraging for remitted depressed people? This study provided preliminary evidence that this might be considered. We tested the idea that in remitted major depression, humor might be an adaptive strategy to regulate negative emotions. We did so by investigating the effects of humorous reappraisal compared with those of positive reappraisal and spontaneous emotion regulation. The results demonstrated that humor required substantial effort from remitted patients, but it was effective in decreasing their negative emotions, increasing positive emotions, and enhancing distance from adversity. Humor was more effective in relation to all measured outcomes than patients’ spontaneous emotion regulation and was similarly as effective positive reappraisal. In view of the fact that positive reappraisal is regarded as one of the most adaptive emotion regulation strategies, our findings seem encouraging; however, we initially predicted that according to experimental nonclinical evidence (Kugler & Kuhbandner, 2015; Samson et al., 2014), humor would predominate over positive reappraisal.

There might be at least two explanations for this finding. One assumes that remitted depressed individuals do not have access to the full advantage of humor because of some cognitive–emotional deficits. Empirical evidence shows that the cognitive processing of humorous materials by depressed people is impaired, mostly because of poor mentalizing and executive performance difficulties (Uekermann et al., 2008), and that it is associated with less intense affective responding (Falkenberg, Jarmuzek et al., 2011a). Although these limitations apply to acute depressive episodes, they may persist in remission, especially when residual symptoms are increased, such as in the case of our sample. If so, the effects of patients’ attempts to humorously reappraise adversity could not possibly go beyond those of nonhumorous positive reappraisal, as these individuals could benefit only from humor's basic mechanisms, which are also the main mechanisms of positive reappraisal (i.e., simple cognitive reframing and elicitation of mild positive affect), and they do not experience dramatic changes in perspective or strong positive emotional responses. Indeed, most participants found their humorous comments only *sort of* amusing and did not respond to them with particularly intense positive emotions.

This result would be consistent with those of previous experimental research on vulnerability to depression, which shows that previously depressed individuals have difficulties in applying more demanding forms of emotion regulation, especially if these involve positive stimuli. Remitted patients were found to exhibit deficits in the processing of positive materials that are activated during a negative mood, thus resulting in the recall of less vivid positivity (Werner‐Seidler & Moulds, 2011). Similarly, some studies have reported that recurrent depression is characterized by cognitive and affective inflexibility (Koval, Kuppens, Allen, & Scheeber, 2012), as well as by negative biases and impaired cognitive control, which lead to the less efficient use of adaptive emotion regulation strategies (see Joormann & D'Avanzato, 2010).

The second explanation concerns the type of nonhumorous reappraisal used in our study; in contrast to previous research based on similar experimental procedures using a serious type of reappraisal (Kugler & Kuhbandner, 2015; Samson et al., 2014), we included a much more effective type, its positive type. Consequently*,* both examined strategies in our study were, in fact, positive forms of cognitive reappraisal, and, thus, they could show similar effects in a single laboratory context. Consensus exists that the effectiveness of emotion regulation strategies depends on the circumstances in which they are used (see Cole & Hollenstein, 2018). Therefore, finding significant differences between different strategies that are generally effective possibly requires distinguishing between the variety of negative situations that could reveal the specific functions of each strategy. For instance, the use of humorous reappraisal in some situations may be perceived as inappropriate, excessively distancing or impossible to generate. Furthermore, humorous reappraisal might be particularly beneficial in uncontrollable situations, in which attempts to solve the problem prove futile and emotional relief may be most helpful. By contrast, positive nonhumorous reappraisal appears more advantageous in controllable situations, as it promotes problem elaboration, which might help overcome the issue and ultimately remove the source of negative emotions.

It should also be stressed that in the present experiment, we instructed participants to produce humorous comments without specifying the exact type of humor, whereas extensive literature indicates that humor can take many forms, and some of these are more adaptive than others (see Martin et al., 2003; Martin, 2007). We analyzed the content of patients’ humorous comments and found that in the vast majority, the positive form of humor was applied, whereas negative humor was used notably less. In view of experimental findings that depressed individuals display negative interpretation biases and difficulties in applying emotion regulation strategies that involve processing mood‐incongruent information (see Joormann & D'Avanzato, 2010), one could expect rather opposite results. Furthermore, nonclinical studies clearly demonstrate that people with increased depressive symptoms habitually use rather negative (maladaptive) than positive (adaptive) components of humor (Kuiper, Grimshaw, Leite, & Kirsh, 2004; Martin, 2007; Rnic et al., 2016).

This result can be partially explained by the social desirability bias, which might have been particularly strong in individuals with residual depressive symptoms, mostly because of their reduced self‐esteem and self‐worth (see Mesmer‐Magnus, Viswesvaran, Deshpande, & Joseph, 2006). Although the participants were assured of anonymity and confidentiality, they probably found it uncomfortable to type negative, aggressive comments on the viewed scenes of tragedy and suffering. Moreover, given that depressed patients are typically preoccupied with negative views of their self and their own situation (Gotlib & Joormann, 2010), the situational context of this study, which concerned the negative situations of other people, possibly did not activate the negative inclinations that could potentially favor negative humor.

It should be stressed that despite the prevalence of the positive form of humor, the consequences of humorous reappraisal were less beneficial than was previously assumed. This seems surprising, as the existing literature suggests that positive, benevolent humor, as opposed to negative, malevolent humor, is highly beneficial for emotional functioning and mental health (see Martin et al., 2003). However, the relationship between the affective tone of humor and its adaptive functions might be more complex in the context of recurrent depression than in other populations. Indeed, experimental evidence shows that individuals with recurrent depression do not take advantage of some positive forms of emotion regulation, such as recalling happy memories (Foland‐Ross, Gilbert, Joormann, & Gotlib, 2015), whereas they can benefit from certain negative strategies, such as suppression (Liverant, Brown, Barlow, & Roemer, 2008). What is more, growing evidence indicates that negative forms of humor can have adaptive effects for some people, particularly those experiencing adverse situations chronically, such as hospital workers (Francis, Monahan, & Berger, 1999) or trauma survivors (Garrick, 2006).

As such, it cannot be excluded that individuals experiencing recurrent episodes of depression also gain more benefits from negative than from positive forms of humor. This idea appears consistent with the long‐established clinical conceptualization of depression as anger turned inward, which is believed to manifest in treating oneself in a hostile way and displaying strong self‐criticism, as opposed to reluctance to criticize others (Vaillant, 2000). In accordance with this view, depressed patients are encouraged to refrain from turning the hostility against the self and to instead express it outwards. Recently, this approach has been strongly represented by emotion‐focused therapy for depression (Greenberg, 2017), which relies on transforming patients’ dysfunctional experiences through expressing them and eliciting the adaptive negative responses, such as adaptive anger.

The present study demonstrated that an important mediator between the use of humor and emotional improvement is distancing, which may suggest that those humor types that create a distant view of distressing events, such as perspective‐taking humor (Lefcourt, 2001) or laughing at oneself (Beermann & Ruch, 2011), might be particularly promising. This idea seems consistent with those of studies on decentring and vulnerability to depression, conducted within the mindfulness‐based approach, indicating that the ability to step back from negative experiences can provide alternative ways of responding, buffer mood deterioration and ultimately prevent relapse (Teasdale et al., 2002).

However, it should be noted that the mechanism of distancing applies only to those types of humor that relate to adverse situations, whereas for depressed people, humorous confrontation with adversity can be over‐demanding. Indeed, reappraising is regarded as more difficult when the negative affect is very strong and when people lack motivation, cognitive resources, or self‐efficacy (see Webb, Lindquist, Jones, Avishai‐Yitshak, & Sheeran, 2018), which, considering the impact of residual depressive symptoms, might be an important impediment. Moreover, finding humor in negative situations may seem incongruous or inappropriate for depressed patients, thus resulting in their reluctance. If so, a simpler form of humor that is unrelated to a negative situation might possibly be more accessible and, additionally, can have stronger effects, as it enables a cutting off from negative materials and diverting all the attention toward amusing stimuli. Although this detached form of humor does not provide a way to reappraise the negative situation and thus cannot activate the mechanism of distancing, its unrelatedness to the stressor and its purely positive content could possibly evoke more intense positive emotions and provide a much stronger distraction from a negative mood.

We also analyzed the potentially adverse effect of failing to produce humor and found that unsuccessful attempts of remitted depressed participants to generate humorous comments had emotional consequences similar to responding to adversity spontaneously, without any risk of failure. This finding seems encouraging, especially in the context of depressed individuals’ susceptibility toward defeat stress (e.g., Metalsky & Joiner, 1993; Taylor, Gooding, Wood, & Tarrier, 2011). However, it can be explained from the perspective of a self‐threat paradigm (Geisler & Weber, 2010), which indicates that humor helps appraise failure as more acceptable and simultaneously enhances positive affect, which may effectively protect one's threatened self‐view. If so, participants’ attempts to generate humorous statements, regardless of the final results, possibly activate self‐serving psychological mechanisms, such as promoting external attribution of defeat, and consequently buffer against mood deterioration.

Several limitations of our study should be noted. First, all dependent variables were analyzed based on self‐reports only. Applying a more multidimensional approach and measure, for instance, psychophysiological parameters, facial expressions, or specific correlates in brain activity, would be more appropriate. Second, we did not control for initial positive and negative emotions prior to the first viewing of negative pictures; depressed individuals can particularly differ in terms of their initial mood state, which could influence the analyzed effects. Third, we assessed patients’ responses at a single point in time rather than observing changes in their emotional responses in the longer term. Fourth, this study included one specific situational context—viewing negative scenes. The extent to which our results can be generalized to other potentially depressogenic situations is therefore unknown. Finally, because of the preliminary nature of the present study, we investigated only one particular type of humor‐based emotion regulation. Compelling evidence demonstrates that distinct components of humor exhibit different relationships with mental health, with some being highly adaptive and others being maladaptive (e.g., Kuiper et al., 2004; Martin, 2007). The emotional tone of humor (positive vs. negative) is just one example of its complex and multifaceted nature. Therefore, more research should be done to analyze different types and forms of humor (e.g., coping humor vs. unrelated joking, nonsense vs. incongruity‐resolution humor), as well as styles of humor (i.e., self‐enhancing, self‐defeating, affiliative, aggressive), and determine their effects in various stress‐provoking contexts.

To conclude, to the best of our knowledge, this is the first study to explicitly introduce the subject of humor in the field of emotion regulation in remitted major depression. We used a well‐proven paradigm and examined a rigorously selected clinical sample of remitted depressed outpatients. The obtained results demonstrated that for individuals at high risk for depression, humor might be an adaptive emotion regulation strategy in dealing with distressing events by reducing negative emotions, enhancing positive emotions, and increasing the distance from adversity. Humor was found to be more effective than regulating negative emotions spontaneously and similarly as beneficial as positive reappraisal, which is one of the most powerful emotion regulation strategies.

Accordingly, the question on whether the use of humor in the face of potentially triggering situations is worth encouraging for remitted individuals can be answered in a complex way that encourages further investigations. On the one hand, humor can be an effective form of emotion regulation for remitted patients, and the risk of failure is relatively minor. On the other hand, humor requires significant effort, and it does not predominate over positive reappraisal. On the basis of these exploratory findings, examining different kinds of humor that are well‐suited to depression vulnerability mechanisms and determining their effects in more specific, self‐relevant contexts are particularly relevant.

## CONFLICT OF INTEREST

None declared.
