# Supplementary material for: Is it worth turning a trigger into a joke? Humor as an emotion regulation strategy in remitted depression
Source: Brain Behav. 2019 Jan 21;9(2):e01213. doi: 10.1002/brb3.1213 (PMC6379593; doi:10.1002/brb3.1213)
Supplement: Supplementary file 1 [file BRB3-9-e01213-s001.pdf]

Supporting Information

- **Procedure outline:**

Recruitment
- psychiatric diagnosis
- clinical interview (SCID I)
- depressive symptoms measurement (BDI II)

Phase I
- watching 28 negative pictures
- after each picture rating positive emotions, negative emotions, distance to adversity (pictures follow randomly)

Phase II
- watching each picture a second time and then a) using humour, b) using positive reappraisal, c) simply viewing.
- rating positive and negative emotions, distance and effort associated with generating the comment (conditions and pictures follow randomly)

Closure
- watching positive pictures
- after each picture, rating positive and negative emotions
- debriefing

- **Experimental procedure (basic elements):**

Phase 1

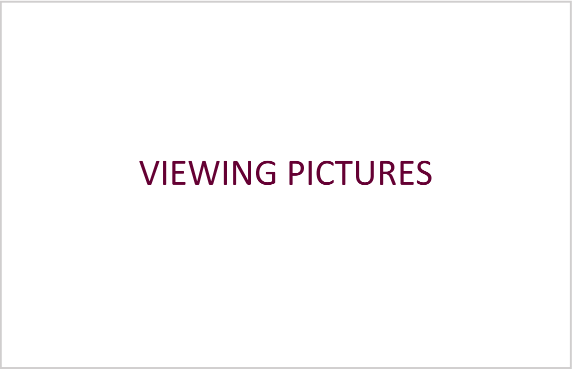

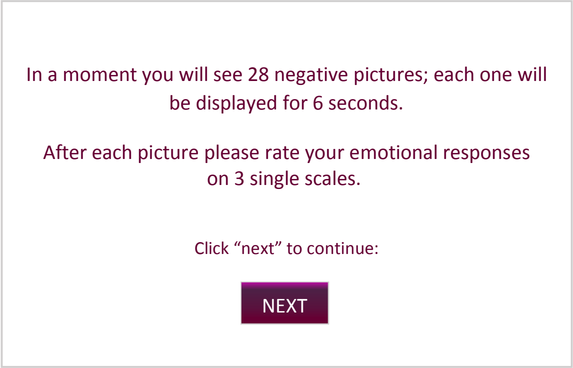

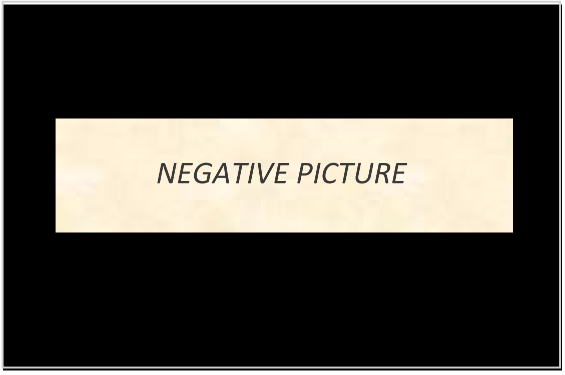

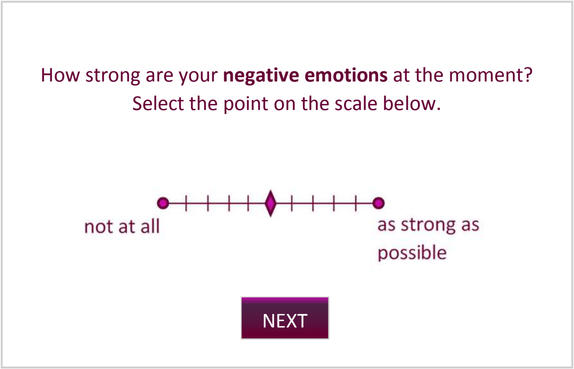

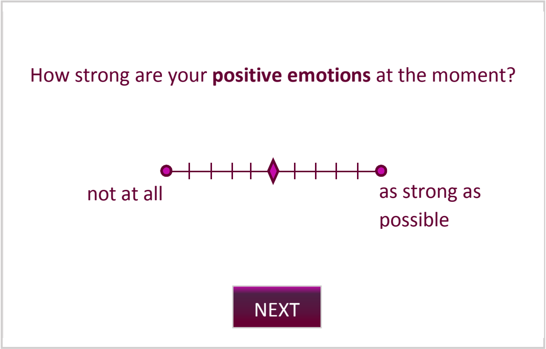

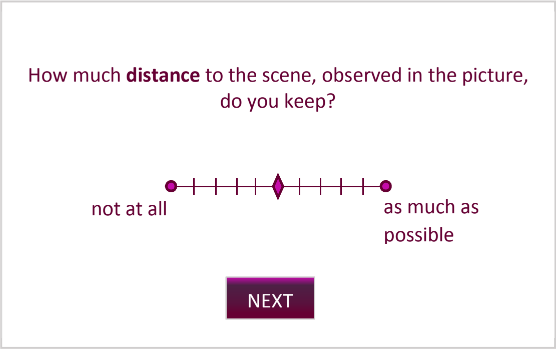

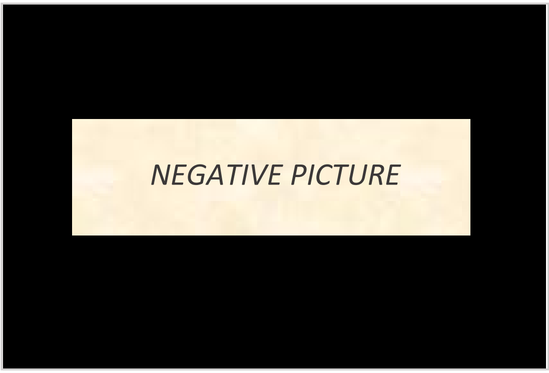

… (28 trials)

Phase 2 – Condition A (humorous reappraisal)

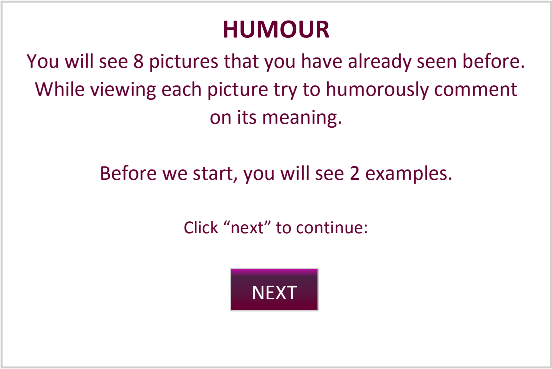

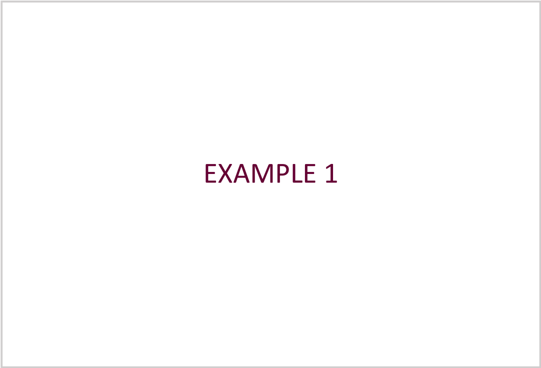

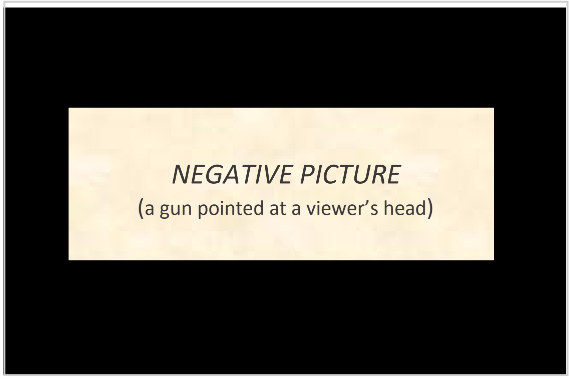

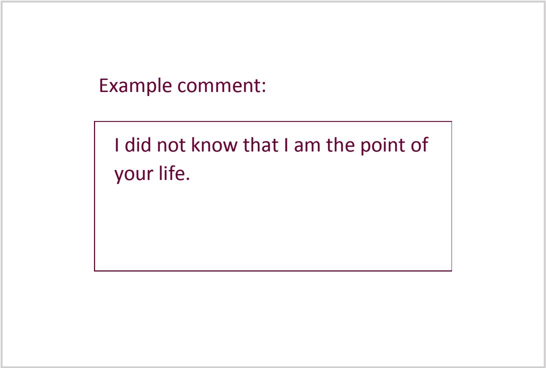

EXAMPLE 2

NEGATIVE PICTURE
(male face with many bruises, dark circles under the eyes, and stiches on the forehead)

Example comment:

And now he's ready to go to the halloween party!

In a moment you will see a picture.

Please, watch it as long as you need and produce a humorous comment.

NEXT

NEGATIVE PICTURE

Have you produced a humorous comment?

YES SORT OF NO

[after "yes" and "sort of" responses]

Please, write your comment below:

Place for your comment...

Click „next" to continue: NEXT

[after "no" response]

Please, write short, why you did not do that:

Place for your explanation...

Click „next" to continue: NEXT

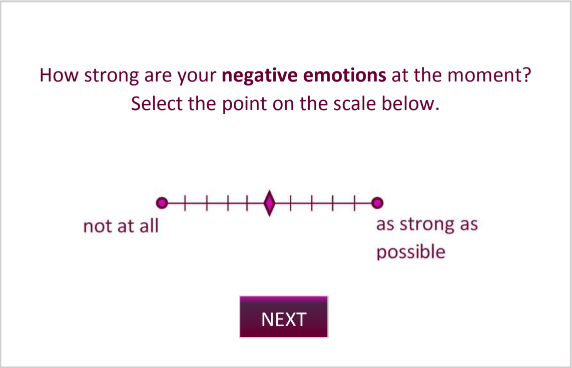

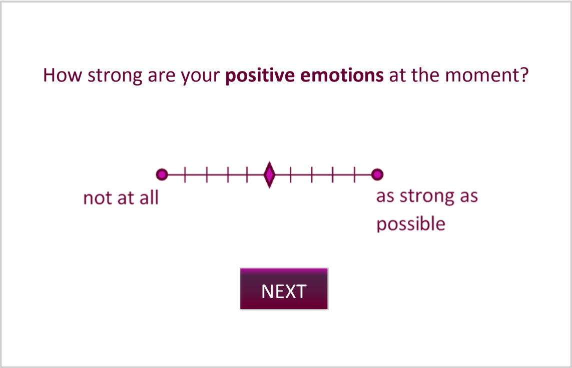

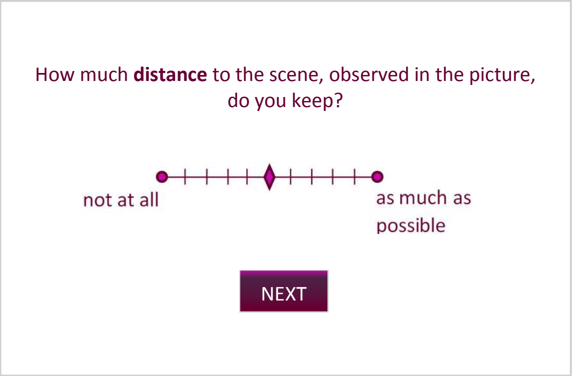

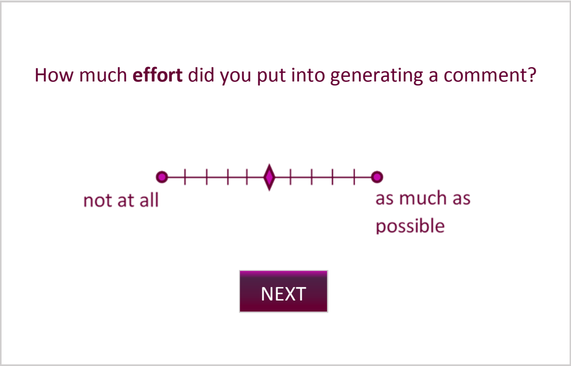

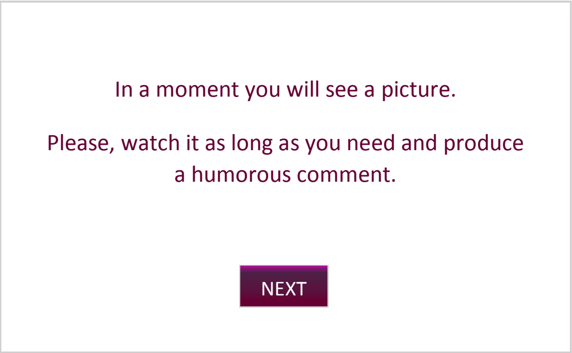

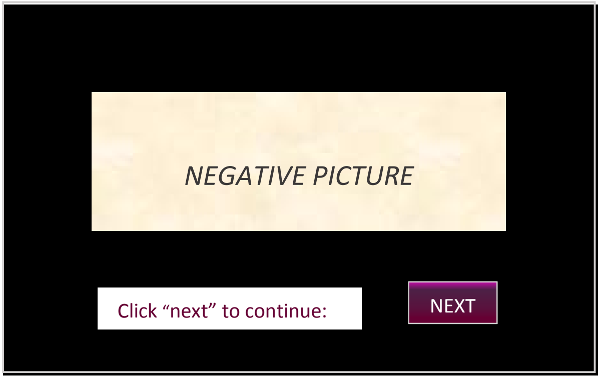

… (8 trials)

Phase 2 – Condition B (positive reappraisal)

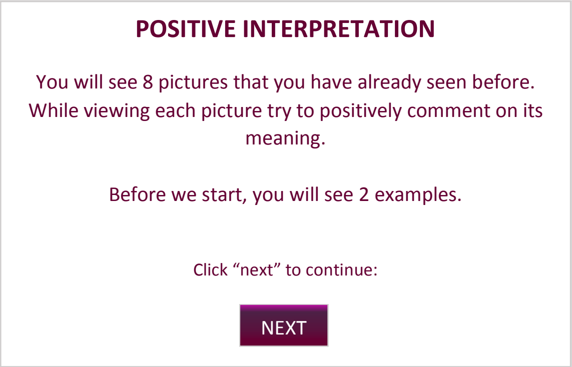

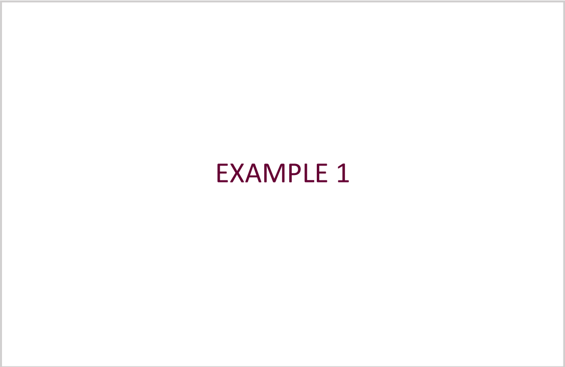

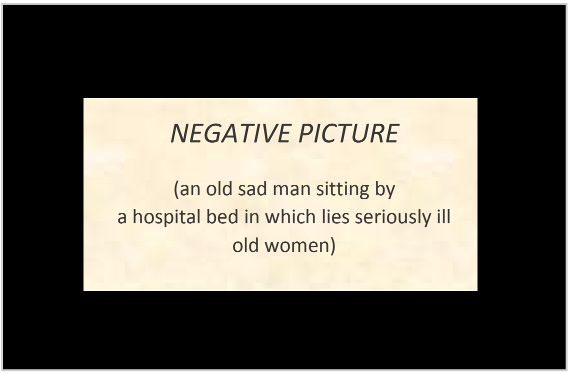
NEGATIVE PICTURE
(an old sad man sitting by a hospital bed in which lies seriously ill old women)

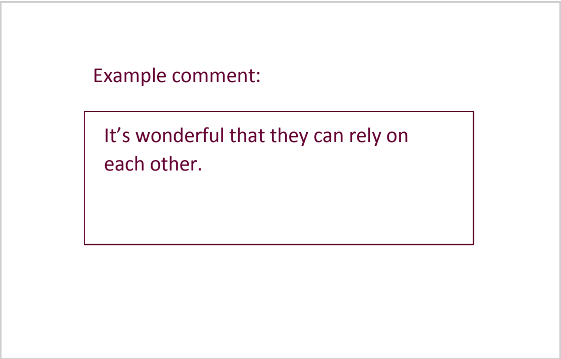
Example comment:
It's wonderful that they can rely on each other.

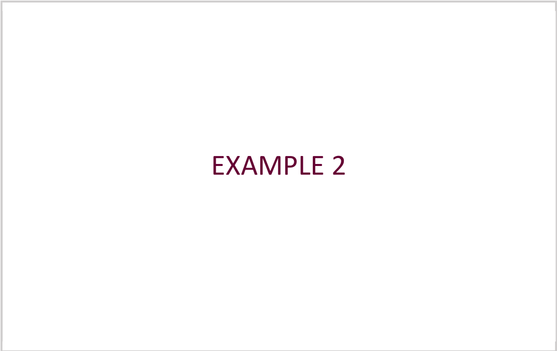
EXAMPLE 2

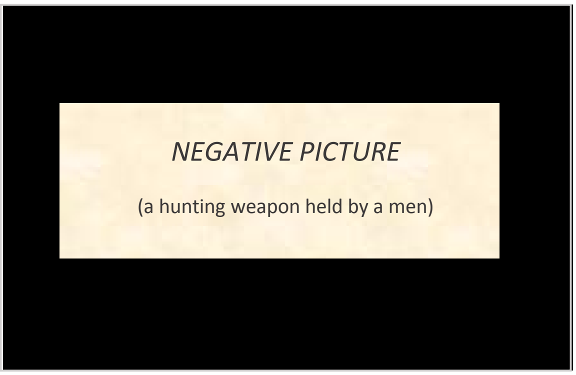
NEGATIVE PICTURE
(a hunting weapon held by a men)

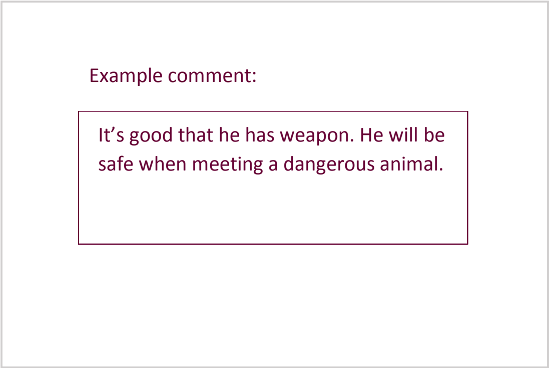
Example comment:
It's good that he has weapon. He will be safe when meeting a dangerous animal.

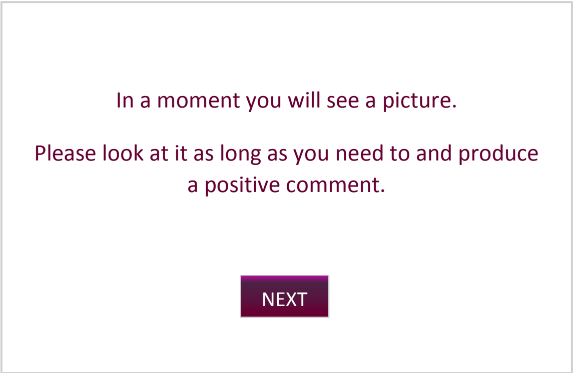
In a moment you will see a picture.
Please look at it as long as you need to and produce a positive comment.
NEXT

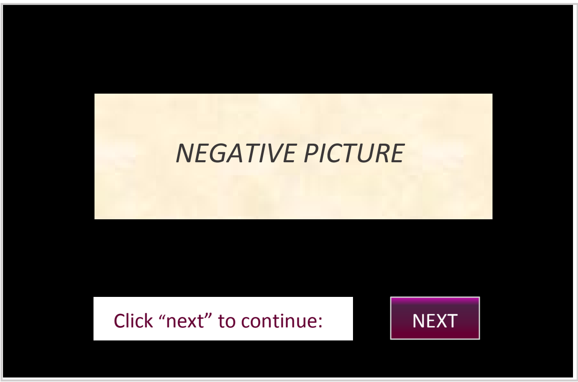
NEGATIVE PICTURE
Click "next" to continue:
NEXT

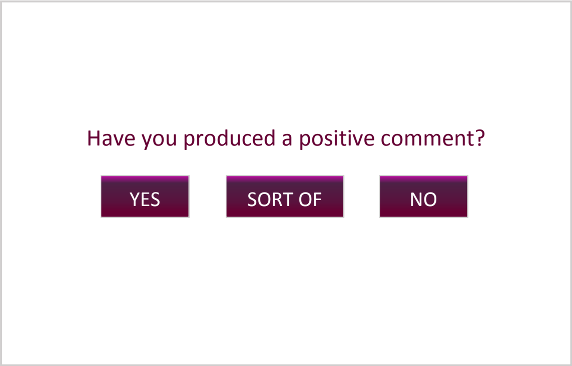
Have you produced a positive comment?
YES
SORT OF
NO

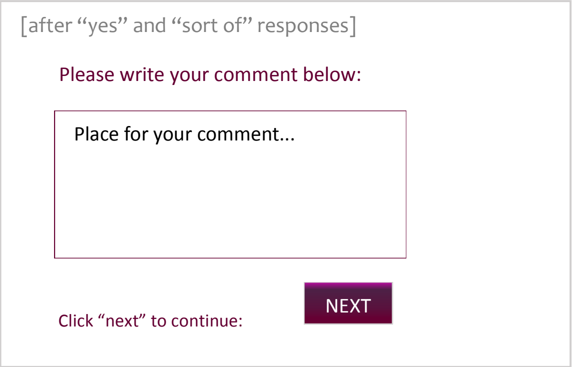
[after “yes” and “sort of” responses]
Please write your comment below:
Place for your comment...
Click “next” to continue:
NEXT

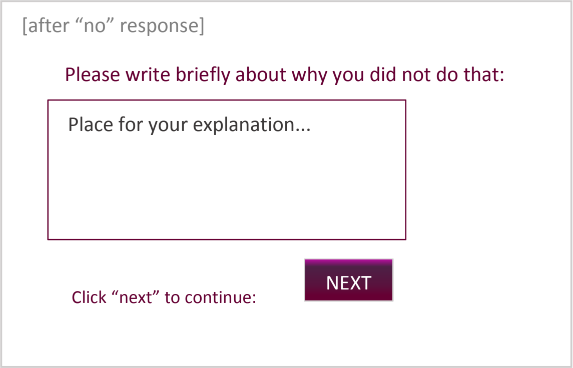
[after “no” response]
Please write briefly about why you did not do that:
Place for your explanation...
Click “next” to continue:
NEXT

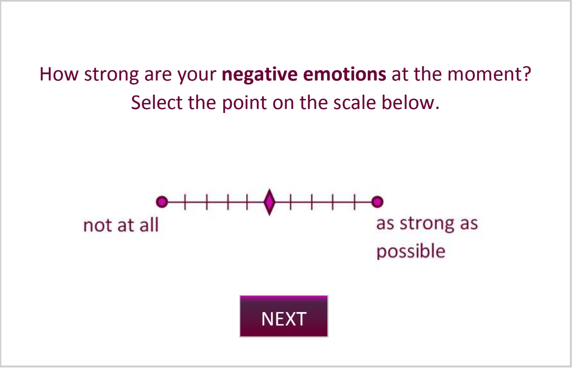
How strong are your negative emotions at the moment? Select the point on the scale below.
not at all
as strong as possible
NEXT

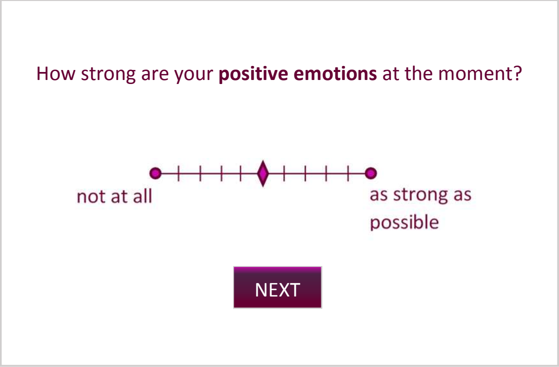
How strong are your positive emotions at the moment?
not at all
as strong as possible
NEXT

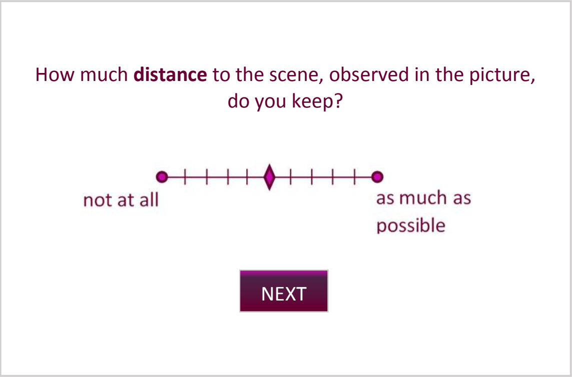
How much distance to the scene, observed in the picture, do you keep?
not at all
as much as possible
NEXT

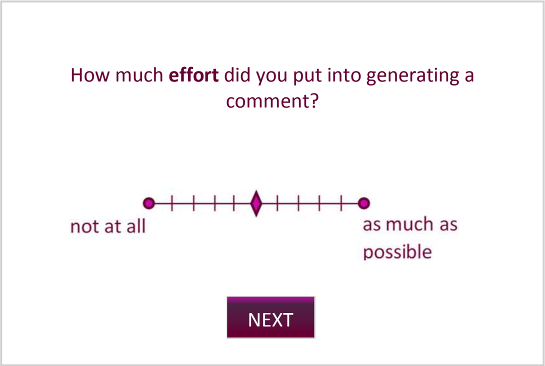
How much effort did you put into generating a comment?
not at all
as much as possible
NEXT

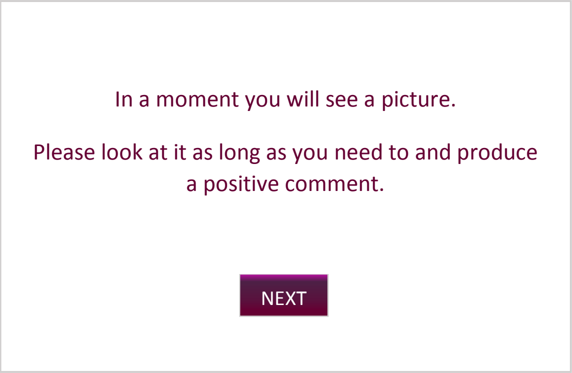
In a moment you will see a picture.
Please look at it as long as you need to and produce a positive comment.
NEXT

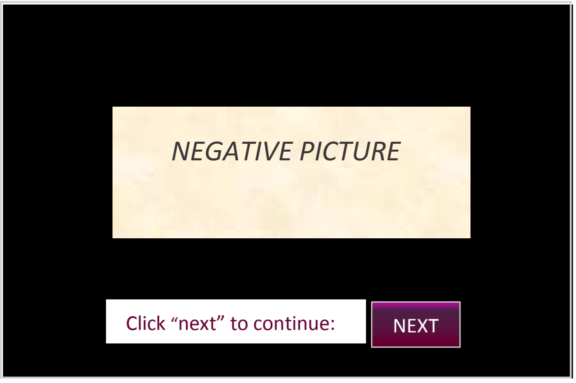
NEGATIVE PICTURE
Click “next” to continue:
NEXT

… (8 trials)

Phase 2 – Condition C (spontaneous emotion regulation)

A SECOND VIEWING

You will see 8 pictures that you have already seen before.
Just watch it; there's no need to do anything else.

Each picture will be displayed for 8 seconds.

Click "next" to continue:

NEXT

In a moment you will see a picture.

Please watch it until it disappears.

NEXT

NEGATIVE PICTURE

What was seen in the picture?
Write 1-2 sentences below:

place for your answer...

Click "next" to continue: NEXT

How strong are your **negative emotions** at the moment?
Select the point on the scale below.

not at all — as strong as possible

NEXT

How strong are your **positive emotions** at the moment?

not at all — as strong as possible

NEXT

How much **distance** to the scene, observed in the picture, do you keep?

not at all — as much as possible

NEXT

In a moment you will see a picture.

Please watch it until it disappears.

NEXT

NEGATIVE PICTURE

… (8 trials)

- ***Closure***

Thank you!

In the end, you will see 9 positive pictures.
Just watch it; there's no need to do anything else.

Each picture will be displayed for 8 seconds.

Click "next" to continue:

NEXT

In a moment you will see a picture.

Please watch it until it disappears.

NEXT

POSITIVE PICTURE

selected from the IAPS database as highly pleasant (ratings 7 – 9), e.g., beautiful nature scenes, little bear cub lying on its mother's back, elderly women laughing and playing with parrots, toddler enjoying watermelon

How strong are your **negative emotions** at the moment?
Select the point on the scale below.

not at all 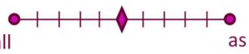 as strong as possible

NEXT

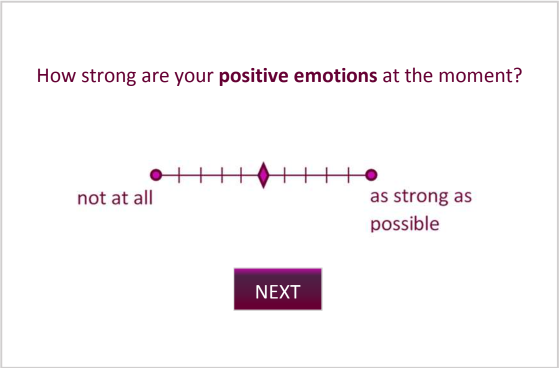
How strong are your **positive emotions** at the moment?
not at all
as strong as possible
NEXT

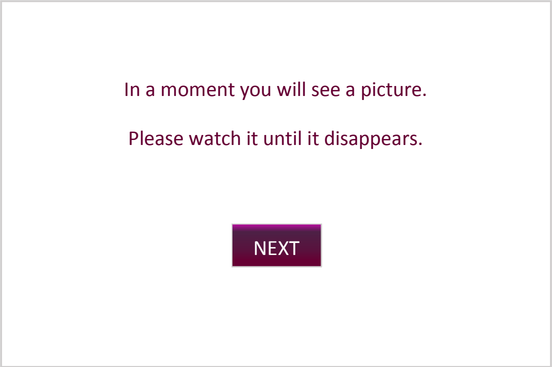
In a moment you will see a picture.
Please watch it until it disappears.
NEXT

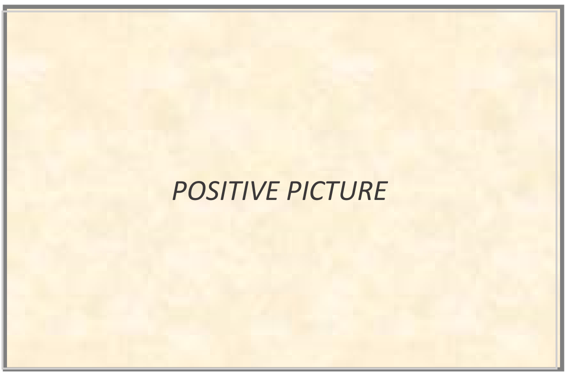
POSITIVE PICTURE

… (9 slides)
